# The reprogramming factor KLF4 in normal and malignant blood cells

**DOI:** 10.3389/fimmu.2025.1584181

**Published:** 2025-06-16

**Authors:** H. Daniel Lacorazza

**Affiliations:** Department of Pathology & Immunology, Baylor College of Medicine, Houston, TX, United States

**Keywords:** KLF4, hematopoietic stem cells, T cells, leukemia, transcription factor

## Abstract

The Krüppel-like factor 4 (KLF4) is an evolutionarily conserved zinc finger transcription factor that regulates cellular processes in stem cells, epithelial cells, and immune blood cells by controlling gene expression through genetic, epigenetic, and chromatin remodeling. The landmark 2006 publication identified KLF4 as one of the factors involved in reprogramming differentiated cells into pluripotent stem cells, sparking increased interest in KLF4 research a decade after its discovery, particularly in the fields of stem cell research, epithelial cell biology, endothelial cell function, and tumorigenesis. Over the years, KLF4 has emerged as a key transcription factor in modulating innate and adaptive immunity, especially in macrophage differentiation and function. This review summarizes the key findings regarding KLF4 in normal blood cells and leukemia.

## Introduction

**Figure 1:**
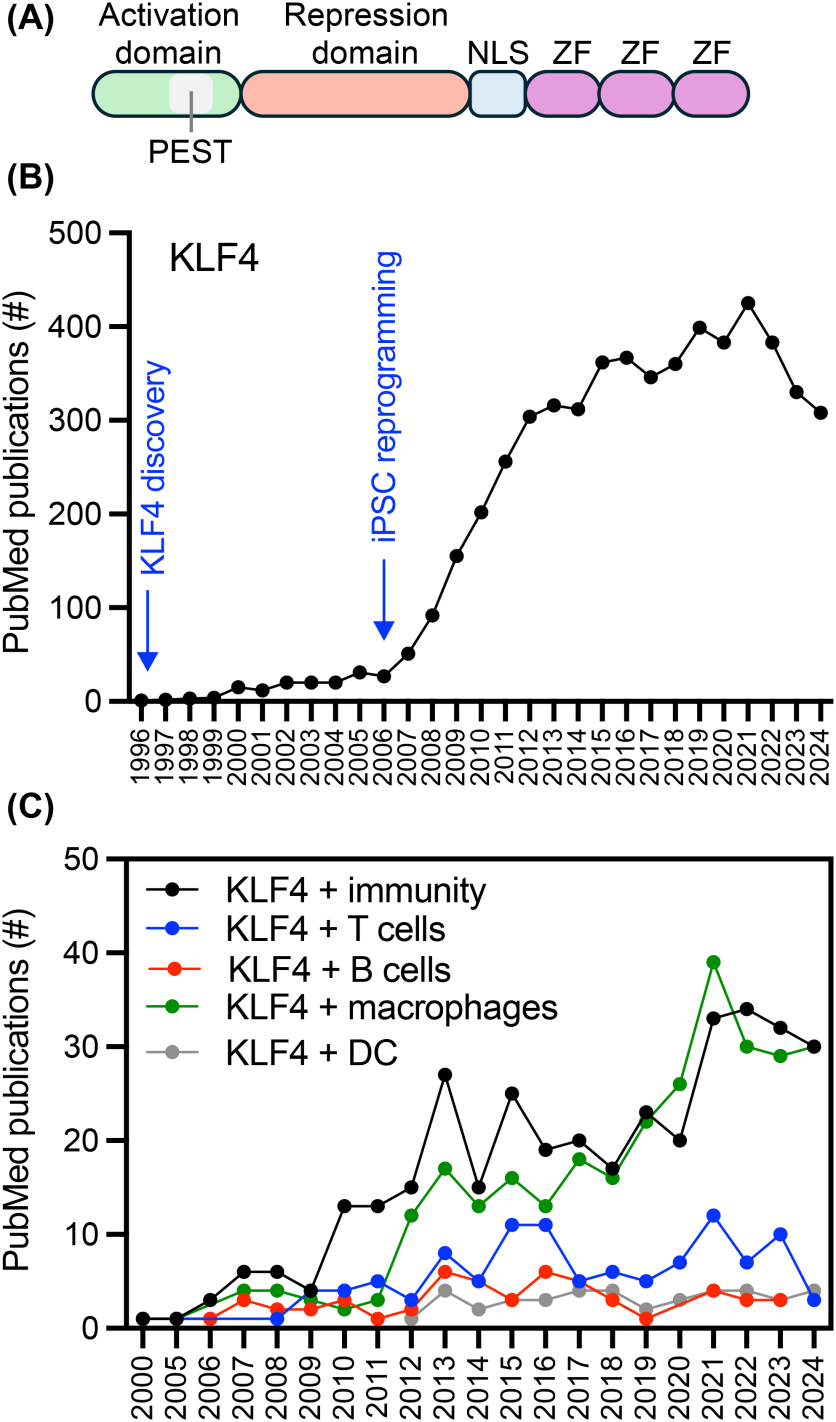
Publication growth related to KLF4 over the years. **(A)** A diagram depicting the main domains in the KLF4 protein. **(B)** Number of publications in PubMed focused on KLF4. **(C)** Number of publications in PubMed focused on KLF4 in conjunction with immunity, T cells, B cells, macrophages, or dendritic cells (DC).

KLF4 is a member of the Krüppel-like factor (KLF) family of transcription factors that play essential roles in stem cell functions, including self-renewal (1–5), pluripotency (1–3, 5, 6), embryogenesis (7), and erythropoiesis (8). The KLF4 protein contains three distinct functional domains involved in DNA binding, gene activation, and gene repression (9). Three zinc fingers within the carboxyl terminal domain mediate the binding of KLF4 to GC-rich sequences (i.e., CACCC) found in gene regulatory promoters and enhancers, leading to the recruitment of co-activators or co-repressors in a cell context-dependent manner (**Figure 1A**) (10). In addition to binding DNA, KLF4 regulates gene expression through protein-to-protein interactions with proteins bound to gene regulatory regions (e.g., the KLF4 to β-catenin interaction regulating the telomerase reverse transcriptase gene) (11). The expression of KLF4 is regulated at the transcriptional level through mechanisms such as CpG methylation, gene regulation, and miRNA, as well as by post-translational modifications including phosphorylation, acetylation, sumoylation, and methylation (12–14). The number of publications on KLF4 has steadily increased since its discovery in 1996, exhibiting an upward trend ten years later when Yamanaka’s group published their groundbreaking findings on reprogramming somatic cells into pluripotent stem cells by the factors KLF4, c-MYC, SOX2, and OCT3/4 (**Figure 1B**) (6). A search of PubMed for KLF4 and specific immune cells reveals that macrophages lead the publications, with a growing interest in T cells (**Figure 1C**).

As a pioneering transcription factor, KLF4 regulates gene expression by binding to silent chromatin and influencing the epigenetic landscape and cell fate (15, 16). To add to the complexity, it has been shown that KLF4 can organize chromatin by forming a liquid-like biomolecular condensate with DNA that recruits OCT4 and SOX2 (17). KLF4 is part of a small group of transcription factors that bind to both unmethylated and CpG-methylated DNA (18, 19). This feature allows KLF4 to bind methylated loci to initiate stem-cell gene expression profiles during reprogramming. KLF factors often work in synchrony. The KLF circuitry composed of KLF2, KLF4, and KLF5 regulates self-renewal in embryonic stem cells (ESC) and the expression of pluripotency genes such as Nanog (1). In addition to ESCs, KLF4 promotes self-renewal in tissue-specific stem cells (e.g., embryo, intestine, skin) and cancer-associated stem cells (1, 13, 20–31).

KLF4 has both tumor suppressor and pro-oncogenic roles in carcinogenesis. This dual role is influenced by several factors, including the cell cycle (e.g., p21 and p53), oncogenic signals (Ras, Wnt, hormone receptors, TGFb, Notch1), and cell survival (32–35). KLF4 tumor suppressor function in solid tumors (e.g., gastrointestinal, lung adenocarcinoma, prostate, pancreatic) and hematological malignancies (e.g., leukemia, lymphoma) has been associated with silencing of the KLF4 locus through different mechanisms (DNA methylation, micro RNAs, histone modifications) (reviewed in (36)). Our group investigated the role of KLF4 in leukemia stem cells using mouse models developed through the retroviral transformation of hematopoietic stem/progenitor cells and transplantation (37–39). In this review article, part of the research topic “*Exploring KLF4’s role in immune cell function and disease progression,”* we will summarize KLF4’s role in blood cells, including immune cells, and focus on hematological malignancies, mainly lymphoid and myeloid leukemias.

## KLF4 regulation of normal blood cells

**Figure 2:**
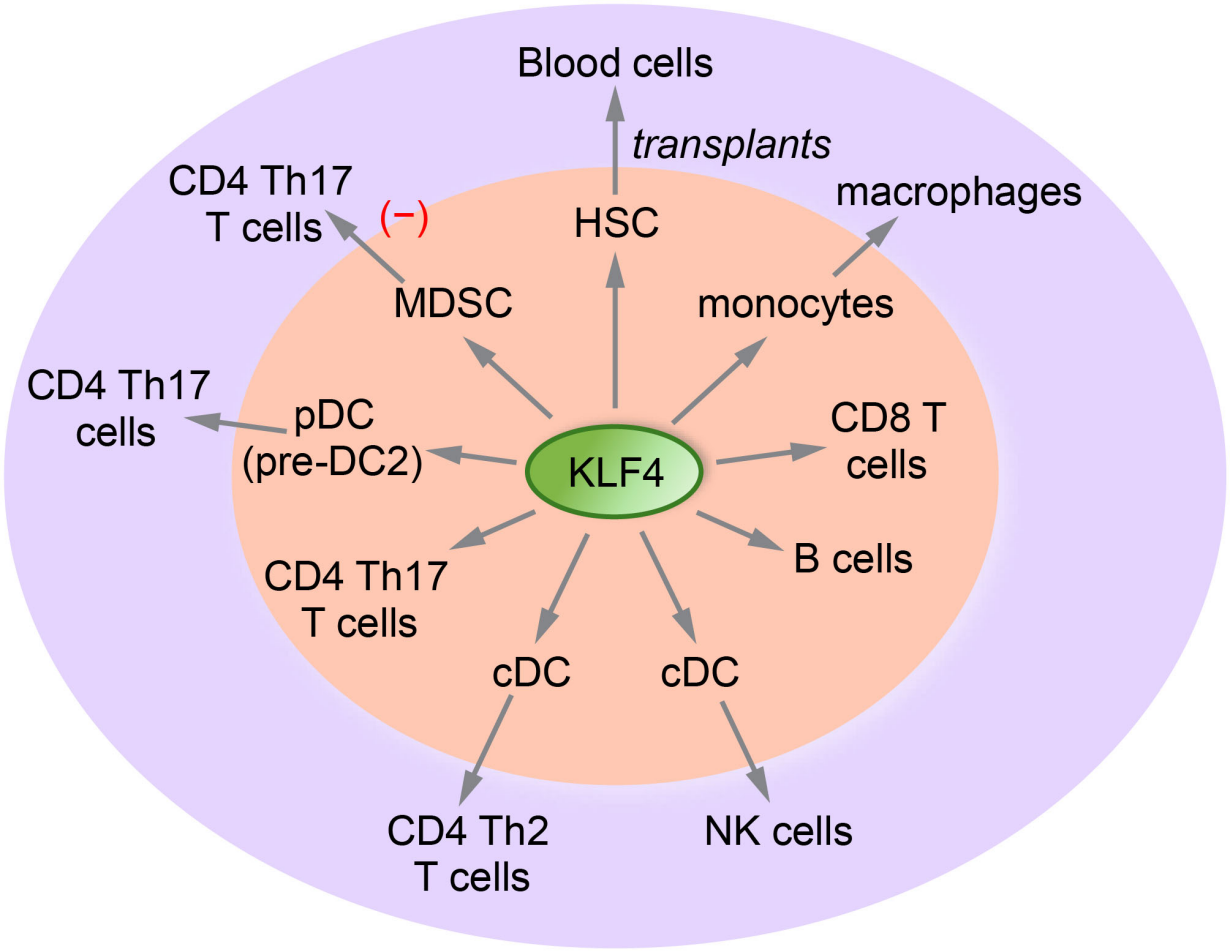
A diagram illustrating the role of KLF4 in immune cells. KLF4 directly regulates the differentiation and function of CD4 and CD8 T cells, conventional dendritic cells (cDCs), B cells, monocytes, and hematopoietic stem cells (HSCs). Additionally, KLF4 secondarily regulates Th17 and Th2 CD4 T cells, NK cells, and macrophages.

KLF4 regulates the function and differentiation of hematopoietic stem cells (HSCs) and mature blood cells, including immune cells (**Figure 2**). It plays a crucial role in monocytic differentiation, macrophage polarization, natural killer cell survival, antibody responses in memory B cells, dendritic cell development, and the inhibition of homeostatic proliferation of naïve T cells (**Table 1**). Next, we will summarize the key findings regarding hematopoietic stem cells and the main immune cells.

**Table 1:** The physiological role of KLF4 in the immune system based on mouse models.

Cell/tissue	Model	Effect	Mechanism	Ref.
Fetal liver HSC	*Klf4* −/− fetal liver chimeras	Normal hematopoietic stem and progenitor cells	n.d.	(40)
Bone marrow HSC	*Klf4* fl/fl VaviCre transplantation	Impaired hematological reconstitution upon transplantation.	KLF4 inhibits TLR4 and NFkB2	(42)
Monocytes Macrophages	Overexpression and knockdown in HL-60 cells and common myeloid progenitor cells *Klf4* −/− fetal liver chimeras *Klf4* fl/fl Mx1-Cre & VaviCre	Alteration monocytic differentiation Monocytic and macrophage differentiation Reduced CD11^+^ Gr1^−^ monocytes in blood	PU.1→ KLF4	(46) (40) (54)
Plasmacytoid dendritic cells (pDC)	*Klf4* fl/fl CD11c-Cre	Defective classical dendritic cell 2 (cDC2) development		(55)
Classical dendritic cells (cDC)	*Klf4* fl/fl Vav1-iCre	Altered development of IRF4-expressing cDCs and impaired Th2 cell responses.	KLF4 → IRF4	(53)
CD8 T cells	*Klf4* fl/fl E8i-Cre *Klf4* fl/fl Mx1-Cre	Impaired differentiation and antitumor function. Increased homeostatic and TCR-mediated proliferation	ELF4 → KLF4 → p21	(66) (63)
Th17 T cells	*Klf4* −/− fetal liver chimeras	Differentiation of Th17 CD4 T cells	KLF4 → IL17	(61)
B cells	*Klf4* fl/fl CD19-Cre	Lower numbers of B cells and proliferation.	KLF4 → Cyclin D2	(68)

Arrows indicate gene activation. n.d., not described.

### Hematopoietic stem cells

**Figure 3:**
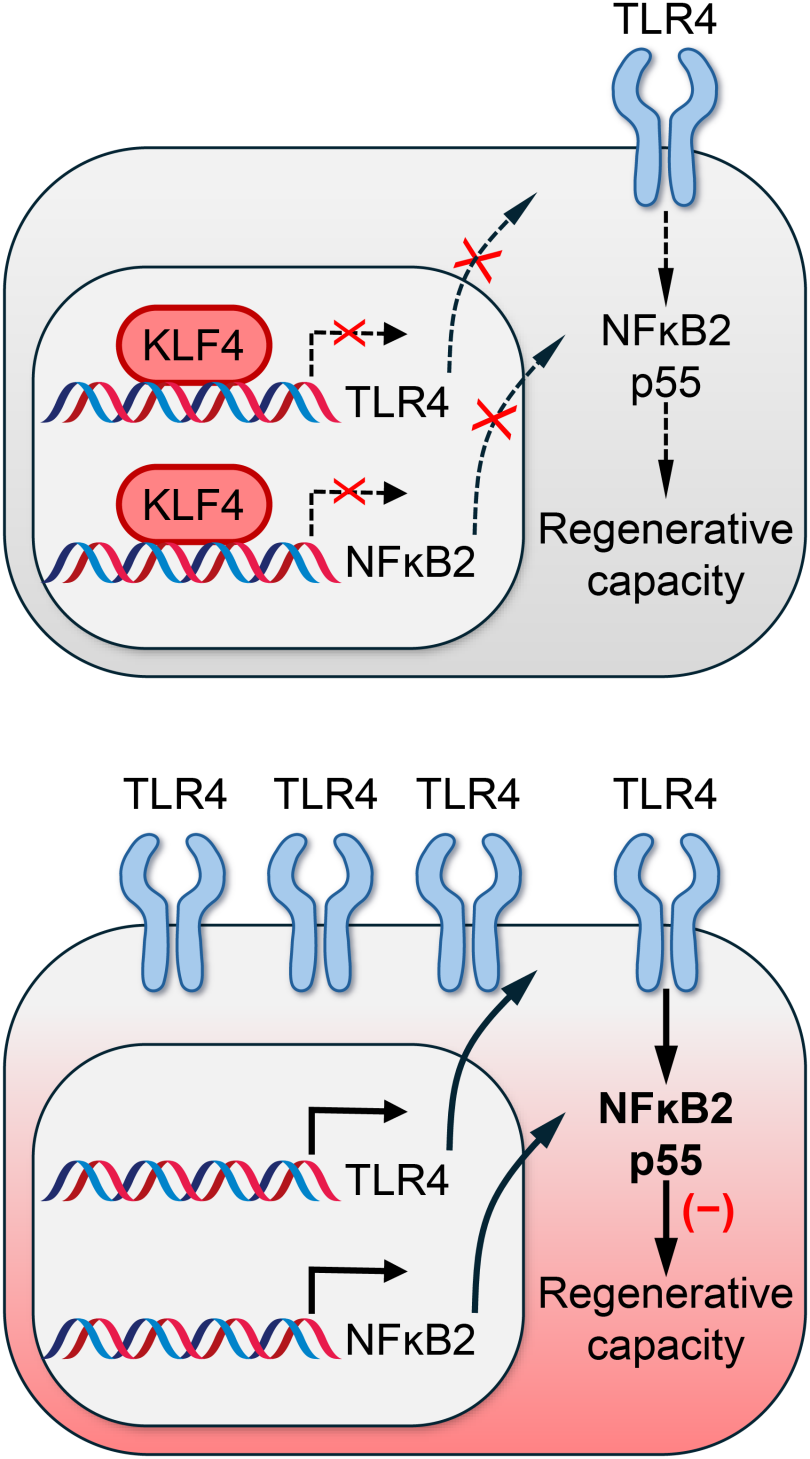
KLF4 protects the regenerative capacity of HSCs. KLF4 represses the expression of TLRs and NFkB2 in HSCs during steady state, preventing chronic inflammation that hinders their capacity to regenerate the hematopoietic system after transplantation into inflamed bone marrow.

An early study demonstrated normal stem cell function using fetal liver (E14.5) *Klf4* ^−/−^ HSCs because the embryonic deletion of the *Klf4* gene leads to perinatal lethality due to an impaired skin barrier (40, 41). Our group recently reported that the conditional deletion of the *Klf4* gene in hematopoietic cells weakens the regenerative capacity of adult HSCs while maintaining many of the stem cell functions during homeostasis (**Figure 3**, **Table 1**) (42). Competitive transplantation of *Klf4* ^fl/fl^ Vav-iCre^+^ HSCs revealed a reduced ability to regenerate the hematopoietic system in an inflamed bone marrow. Transcriptome analysis revealed that loss of KLF4 was linked to increased expression of toll-like receptors (TLRs), such as TLR4, and the activation of the non-canonical NFκB2 (nuclear factor kappa light chain enhancer of activated B cells) pathway (**Figure 3**) (42). This finding aligns with earlier studies showing that chronic activation of the NFκB pathway causes bone marrow failure by disrupting the quiescence and impairing the regenerative function of HSCs (43). Activating NF-κB via the transgenic expression of constitutively active IKK2, enzyme that activates NF-κB, promotes HSC proliferation, decreases quiescence, and impairs the repopulating ability in secondary and competitive transplants (44). Thus, KLF4 preserves the capacity of HSCs to regenerate blood cells during transplantation-induced hematopoiesis by suppressing the expression and activity of toll-like receptors (TLRs) during homeostasis. However, one question remains unanswered: does KLF4 activity decline with age in HSCs, contributing to chronic stem cell inflammation and potentially linking to stem cell aging and myelodysplasia?

### Monocytes and macrophages

The role of KLF4 in inflammatory diseases has been extensively studied (45). This review will briefly summarize the role of KLF4 in differentiating these cells, focusing on other myeloid and lymphoid cells. Research on loss- and gain-of-function in HL-60 cells and murine stem/progenitor cells has established the role of KLF4 in differentiation toward the monocytic lineage (46). Furthermore, ectopic expression of KLF4 in PU.1-null fetal liver cells restored the ability to differentiate into monocytes, suggesting that PU.1 promotes KLF4 expression during monocyte differentiation. This finding was confirmed by the transplantation of fetal liver *Klf4* ^−/−^ HSCs, which showed impaired differentiation of monocytes (40). Research has demonstrated that interferon regulatory factor 8 (IRF8) induces the expression of the *Klf4* gene in myeloid progenitor cells, while PU.1, a crucial transcription factor in myeloid development, targets KLF4 (46, 47). KLF4 also regulates the differentiation of monocytes into macrophages and tumor-associated macrophages during tissue migration (48–50). KLF4 is essential for M1-type differentiation and influences macrophage activation, activating cytokine response (40, 46, 47, 51). Interestingly, decreased diurnal KLF4 expression in aged macrophages disrupted diurnal rhythms in phagocytic activity, indicating that KLF4 is involved in the circadian regulation of the innate immune response during aging (52). This finding raises the concern that many functional studies of KLF4 in different blood cells may need to be re-evaluated to investigate the impact of circadian variations on KLF4 expression.

### Dendritic cells

Conditional *Klf4* gene deletion through Vav-iCre transgenic mice showed loss of Ly6C^hi^ monocytes and reduced interferon regulatory factor 4 (IRF4) expression on pre-conventional dendritic cells (pre-cDC) but not mature cDCs (53). The splenic classical DCs (cDC: CD11c^hi^ CD11b^+^), regulated by KLF4, enhance the survival of NK cells in peripheral tissues through IL-15 signaling (54). Conditional *Klf4* deletion in cDCs using Itgax-Cre mice impairs Th2 cell responses to the helminth *Schistosoma mansoni* (53). Additionally, KLF4 is essential for differentiating a subpopulation of plasmacytoid DCs, pre-DC2 cells, into CX3CR1^+^ ESAM^−^ (cDC2b) cells, which play a key role in maintaining the Th17 cell pool in the surveillant lymph nodes of the skin (55).

### Natural killer cells

Natural killer (NK) cells are crucial in the innate immune response. KLF4 regulates the differentiation and survival of NK cells through several mechanisms. KLF4 enhances the survival of NK cells in peripheral tissues by promoting the differentiation of splenic conventional dendritic cells (cDC) defined as CD11chi CD11b+ cells (54). KLF4 induces the expression of the NKG2D ligand MICA in acute myeloid leukemia cell lines; however, the function of KLF4-NKG2D in primary leukemic cells still requires additional investigation (56). KLF4 induces ICAM-1 expression in hypoxia-sensitive epididymal cells via the KLF4-ASH1L-ICAM-1 axis, which leads to NK cell activation and epididymal damage (57). KLF4 interacts with ASH1L, a subunit of the histone methyltransferase complex MLL, bringing it to the adhesion molecule ICAM-1 promoter for the tri-methylation of histone H3 at lysine 4 (H3K4me3), an epigenetic mark linked to gene activation.

### T cells

T cells are developed in the thymus from bone marrow-derived T cell progenitor cells. Early thymic progenitors do not express CD4 or CD8 (double negative: DN) and can be further classified into subsets based on CD44 and CD25 expression, progressing in differentiation from DN1 (CD44^+^ CD25^−^) to DN2 (CD44^+^ CD25^+^) to DN3 (CD44^−^ CD25^+^) and finally to DN4 (CD44^−^ CD25^−^) cells, with the latter differentiating into immature double-positive (DP) cells that finally mature in either CD4 or CD8 T cells. The expression of Yamanaka factors in thymocyte subsets shows that KLF4 is highest in embryonic stem cells, with lower levels in HSCs, CLPs, and DN1, and continuing to drop in DN2-DN4 and DP cells (58). The conditional deletion of the *Klf4* gene in hematopoietic cells using Vav-iCre transgenic mice leads to decreased cellularity across all thymocyte subsets, from DN1 to CD4 and CD8 single-positive cells, despite showing no significant differences in cell proliferation and survival in peripheral tissues, which are regulated by other mechanisms (59). The expression of KLF4 in thymic endothelial cells (TEC) during late pregnancy prompted the conditional deletion of the *Klf4* gene in these cells. This deletion led to a notable reduction in thymic size and cellularity during pregnancy-induced thymic involution despite causing minimal changes in thymic cellularity during homeostasis (60). This finding suggests that KLF4 preserves the integrity of thymic endothelial cells during pregnancy and thymic regeneration after childbirth.

As stated above, KLF4 can modulate the function of different T cell subsets by promoting the differentiation of specialized dendritic cells or myeloid-derived suppressor cells. KLF4 promotes Th17 differentiation in CD4 T cells by activating the IL17 promoter (61). On the other hand, KLF4 inhibits Th17 differentiation in the ob/ob mouse pressure ulcer model, which promotes diabetic chronic wound healing through myeloid-derived suppressor cells (62). In naïve CD8 T cells, the transcription factor ELF4 directly activates the *Klf4* gene, inhibiting cell division through cell cycle kinase inhibitor p21 expression during homeostasis and in response to antigen-driven proliferation by activating the T cell receptor (TCR) (63). CD8^+^ T cells from mice with *Klf4* gene deletion, induced by the Mx1-Cre (cre-recombinase driven by the Mx1 promoter) and poly-I:C injection (double stranded RNA induces systemic IFN*γ* secretion) model, showed increased cell division upon *in vitro* crosslinking with anti-CD3 and anti-CD28, alongside homeostatic expansion of CD8^+^ T cells showing a memory-like immunophenotype (CD122^+^ CD44^hi^) (63). *Klf4*-null CD8^+^ T cells expressing the OT1 transgene (ovalbumin-specific TCR) demonstrated enhanced expansion in both primary and recall responses to infection with *Listeria monocytogenes*-OVA (bacteria expressing ovalbumin) (59). The regulation of KLF4 by ELF4 was governed upstream by the ERK and mTOR pathways in CD8^+^ T cells (64). Consistent with these findings, the proteasomal degradation of KLF4, which is ubiquitinated at lysine by the E3 ligase Mule, promotes the transition from G1 to S-phase in T cells (51). As a result, deleting the *Klf4* gene exacerbates experimental autoimmune encephalomyelitis due to the pathogenic role of Th17 cells while hindering the clearance of lymphocytic choriomeningitis virus (LCMV) infection (65). A group reported that KLF4 is a hallmark of cytolytic effector-like CD8 T cells during the exhaustion process; therefore, ectopic KLF4 expression can enhance the activity of exhausted T cells and is associated with better prognosis in cancer patients (66). Stabilization of the KLF4 protein through PRMT5 arginine methylation, which prevents ubiquitination by VHL, contributes to genome stability and carcinogenesis (12); however, the role of this post-translational modification in regulating homeostatic and antigen-driven T cell proliferation remains unexplored.

### B cells

Transcriptional profiling of multipotent progenitor cells induced to differentiate into B cells by Id3 expression revealed a wave of priming transcription factors (e.g., KLF4, NR4A2, EGR1) before the expression of core transcription factors E2A, EBF1, and PAX5 (67). The conditional deletion of *Klf4* in B cells using the CD19-Cre system reduces the frequency of pro-B and mature B cells and lowers proliferation induced by crosslinking with anti-IgM and anti-CD40, attributed to the regulation of the cyclin D2 promoter (68). Transcriptional analysis during *in vitro* B cell differentiation identified KLF4 as one of the transcription factors involved in the early priming of B cell progenitor cells (67). KLF4 expression in bone marrow plasma cells promotes a gene expression profile supporting early cell differentiation (69). Naïve B cells express higher levels of KLF4, KLF9, and PLZF compared to memory B cells, and this expression decreases following B cell activation. This indicates that the reduced expression in memory B cells allows them to enter the cell cycle rapidly, which is a key feature of memory cells (70). B cells can undergo reprogramming; a tetracistronic Sendai virus carrying OCT4, SOX2, KLF4, and MYC can reprogram CD19-positive B cells from cord blood or peripheral blood into induced pluripotent stem cells, which are extremely useful for studying B cell function and transformation in hematological malignancies (71).

## KLF4 has tumor suppressive and pro-leukemic functions in leukemia

**Figure 4:**
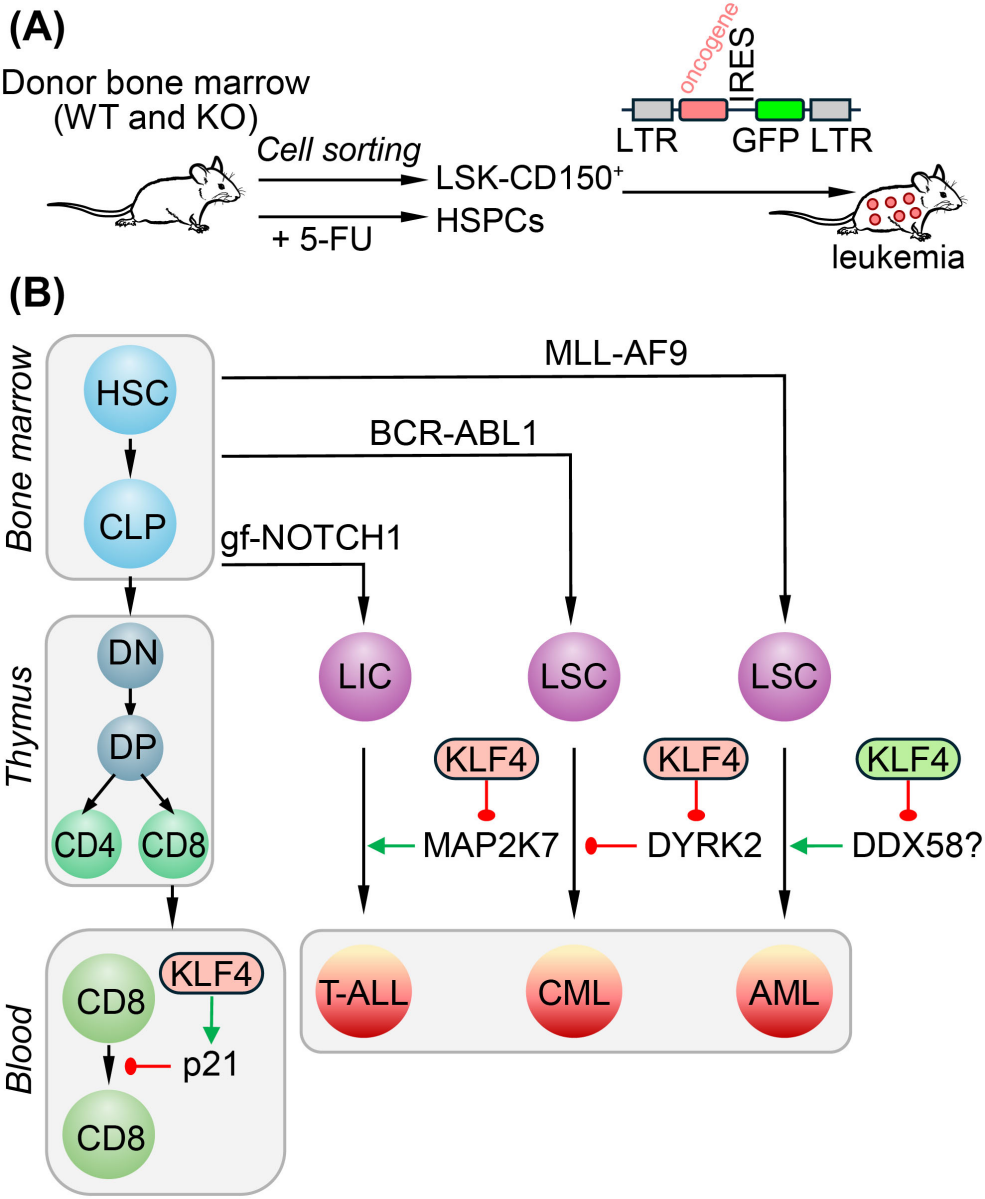
Control of leukemia stem cells (LSC) and leukemia-initiating cells (LIC) in myeloid and lymphoid leukemia. **(A)** Transduction of hematopoietic stem/progenitor cells with a retrovirus carrying an oncogene: BCR-ABL1 to induce chronic myeloid leukemia (CML), gain-of-function NOTCH1 mutant (gfNOTCH1) for T-cell acute lymphoblastic leukemia (T-ALL), and MLL-AF9 for acute myeloid leukemia (AML). **(B)** KLF4 represses MAP2K7 in T-ALL (tumor suppressor function). KLF4 exhibits a pro-oncogenic function in BCR-ABL1-induced CML by repressing the DYRK2 gene, preventing inhibition of LSC self-renewal. In AML, the upregulation of DDX58 in *Klf4* knockout LSCs does not appear to contribute to the impaired frequency of LSCs. The activity repressing or activating target gene expression is indicated in red and green, respectively.

Most research on KLF4 in cancer centers compares its expression in patient samples with that in healthy individuals. Examining the correlation between DNA methylation and gene expression revealed that hypermethylation of the KLF4 gene was associated with lower KLF4 expression in fifteen patients with chronic lymphocytic leukemia (72). However, DNA methylation profiling in leukemia does not have diagnostic value, and its potential link to tumor suppression needs to be investigated in mouse models. In B-cell non-Hodgkin lymphoma, KLF4, regulated by the transcription factor YY1, acts as a tumor suppressor by inducing apoptosis through the pro-apoptotic gene BAK1 (36, 73, 74). Research on the oncogenic role of KLF4 has mainly focused on overexpression in established cell lines, with limited assessment in mouse models of cancer, especially concerning blood malignancies. In this review, we will summarize studies from our group that utilized conditional *Klf4* deletion and retroviral transduction models with oncogenes to investigate their role in leukemia stem cells (LSC) within lymphoid and myeloid leukemia (**Figure 4A**). Generally, purified HSCs (Lin− Sca-1+ c-kit+ CD150+= LSK-CD150 cells) or bone marrow cells from mice pre-treated with 5-fluorouracil (5-FU) to enrich bone marrow in hematopoietic stem/progenitor cells (HSPC) are utilized for retroviral transduction carrying leukemia specific oncogenes (**Figure 4A**). Retrovirus carrying a gain-of-function NOTCH1 mutant, the constitutively activated BCR-ABL1 kinase, or the fusion MLL-AF9 are used to induce T-cell acute lymphoblastic leukemia (T-ALL, chronic myeloid leukemia (CML), or acute myeloid leukemia (AML), respectively.

Acute lymphoblastic leukemia (ALL) is the most common cancer in children under 14 years old, with T-cell ALL (T-ALL) being a subtype recognized for its high relapse rate. KLF4 in T-ALL has been studied due to its ability to inhibit the proliferation of naïve T cells and its suppressive role in T-ALL cell lines (37, 75, 76). A gene expression profiling analysis in pediatric leukemia indicates that KLF4 is significantly downregulated in T-ALL compared to normal bone marrow, particularly in T-ALL subtypes associated with the worst prognosis (37). This finding aligns with the epigenetic silencing of the KLF4 gene due to DNA CpG methylation seen in children with T-ALL, which was not present in the bone marrow and T cells obtained from healthy individuals (37). The conditional deletion of the *Klf4* gene accelerated leukemia in the NOTCH1-induced T-ALL mouse model, increasing both the proliferation of T-ALL cells and the frequency of leukemia-initiating cells (LIC) measured in a limiting dilution transplantation study (37). Since KLF4 represses the *Map2k7* gene, which encodes a dual specificity mitogen-activated protein kinase kinase 7 (MAP2K7), the epigenetic silencing of KLF4 in patients and its conditional deletion in the mouse model of T-ALL lead to the aberrant activation of the MAP2K7-JNK pathway (**Figure 4B**) (37, 77). Pharmacological inhibition of the MAP2K7 kinase has demonstrated anti-leukemic effects in T-ALL cell lines and patient-derived xenograft cells (78–80). In summary, KLF4 has tumor suppressor activity in pediatric T-ALL, at least in part by inhibiting MAP2K7, which may be considered for therapeutic targeting. Research now is focused on developing specific, potent, and safe MAP2K7 inhibitors to translate into the clinics.

Chronic myeloid leukemia (CML) is caused by the oncoprotein BCR-ABL1, which is the product of the chromosomal translocation t (9, 22). While remission can be achieved with the tyrosine kinase inhibitor Imatinib, patients must be kept on treatment for life. As a result, a considerable amount of research focuses on LSCs due to their resistance to Imatinib and potential to cause relapses if the treatment is stopped. Using the retroviral BCR-ABL1 model to induce myeloproliferative-like disease, which serves as a model for CML, researchers found that the conditional deletion of the *Klf4* gene prolonged overall survival. This effect is attributed to the inhibition of self-renewal and the induction of apoptosis in leukemia stem cells (LSCs), a rare population of leukemic cells with stem cell features that continuously feed the neoplasm (38). KLF4 loss leads to the upregulation of the dual-specificity DYRK2 kinase since KLF4 represses the expression of the *Dyrk2* gene (**Figure 4B**). DYRK2 upregulation was linked to increased apoptosis through p53 phosphorylation and c-Myc proteasomal degradation via prime-phosphorylation, as DYRK2 can be activated by auto-phosphorylation (38). In addition to the genetic upregulation of DYRK2, inhibiting the ubiquitin ligase SIAH2, which mediates the proteasomal degradation of DYRK2, with synthetic vitamin K3 stabilizes the DYRK2 protein (38). However, vitamin K3 cannot be administered to patients due to its high toxicity. Like embryonic stem cells, KLF4 promotes self-renewal in CML LSCs, but it does so by repressing a DYRK2-mediated inhibition mechanism. It needs to be further investigated whether the pharmacological stabilization of DYRK2 protein could help achieve treatment-free remission by eliminating LSCs.

Acute myeloid leukemia (AML) is an aggressive cancer that primarily affects the elderly and has a poor prognosis due to ineffective treatments. Unlike T-ALL, the *KLF4* gene is not silenced epigenetically by CpG methylation in AML. Genome editing of the *KLF4* gene in the AML cell lines NB4 and MonoMac6 by CRISPR/Cas9 showed reduced cell growth and increased apoptosis (81). In the AML model induced by retroviral expression of the MLL-AF9 fusion in hematopoietic stem/progenitor cells followed by transplantation, the loss of KLF4 caused improved survival of leukemic mice, which was linked to a reduced frequency of LSCs identified in this model as granulocyte monocyte progenitor (GMP) cells that are positive for MLL-AF9 (**Figure 4B**) (39, 81). Gene expression profiles obtained from purified leukemic GMP cells of wild-type and *Klf4* knockout leukemic mice indicate that the loss of KLF4 is associated with decreased expression of genes regulated by MLL-AF9, as well as a leukemic stemness gene signature and cell cycle regulators (39). Genes related to inflammation, such as the dsRNA helicase DDX58, were upregulated in murine HSCs and LSCs (L-GMP), indicating a role in the inflammatory type I interferon pathway in AML. However, experiments of knocking down DDX58 in *Klf4* knockout MLL-AF9-induced leukemia suggested that elevated levels of DDX58 in *Klf4* knockout LSCs did not contribute to impaired LSC frequency despite reducing clonogenicity in methylcellulose (39). Overall, KLF4 supports MLL-AF9-driven AML by sustaining the expression of genes related to LSC stemness and MLL target genes.

## Concluding remarks

Recently, there has been a growing interest in studying KLF4 in normal and malignant hematopoiesis. In normal hematopoietic stem cells, KLF4 preserves their regenerative capacity after transplantation by repressing TLR and NFkB2 during homeostasis. In leukemia, KLF4 may inhibit or promote self-renewal in leukemic stem cells, depending on oncogenic signals and KLF4’s dual role as a transcriptional activator and repressor. Likewise, KLF4 can function as either a tumor suppressor or a pro-oncogene, depending on the regulation of cell cycle and signaling within an oncogenic environment. In myeloid leukemias, KLF4 has pro-leukemic function in CML by suppressing a mechanism that inhibits LSC self-renewal, and in AML by promoting the expansion of LSCs. In line with its role in inhibiting cell division in normal T cells, KLF4 functions as a tumor suppressor in leukemic T cells by repressing a kinase that drives T-ALL cell proliferation. Further investigation is needed to clarify the physiological and pathological roles of KLF4 in various blood lineages and to identify actionable target genes and downstream mechanisms for potential pharmacological intervention. This is necessary because transcription factors are often considered undruggable, and there are concerns regarding systemic therapy targeting KLF4, given its dual carcinogenic functions.
